# Crafting Care That Fits: Workload and Capacity Assessments Complementing Decision Aids in Implementing Shared Decision Making

**DOI:** 10.2196/13763

**Published:** 2020-03-25

**Authors:** Thomas H Wieringa, Manuel F Sanchez-Herrera, Nataly R Espinoza, Viet-Thi Tran, Kasey Boehmer

**Affiliations:** 1 Department of Medical Psychology Amsterdam UMC Amsterdam Netherlands; 2 Knowledge and Evaluation Research Unit Mayo Clinic Rochester, MN United States; 3 METHODS Team Centre of Research in Epidemiology and StatisticS Université de Paris, Institut National de la Santé et de la Recherche Médicale Paris France

**Keywords:** decision making, decision support techniques, patient-centered care

## Abstract

About 42% of adults have one or more chronic conditions and 23% have multiple chronic conditions. The coordination and integration of services for the management of patients living with multimorbidity is important for care to be efficient, safe, and less burdensome. Minimally disruptive medicine may optimize this coordination and integration. It is a patient-centered approach to care that focuses on achieving patient goals for life and health by seeking care strategies that fit a patient’s context and are minimally disruptive and maximally supportive. The cumulative complexity model practically orients minimally disruptive medicine–based care. In this model, the patient workload-capacity imbalance is the central mechanism driving patient complexity. These elements should be accounted for when making decisions for patients with chronic conditions. Therefore, in addition to decision aids, which may guide shared decision making, we propose to discuss and clarify a potential workload-capacity imbalance.

## Minimally Disruptive Medicine

About 42% of adults have one or more chronic conditions and 23% have multiple chronic conditions (ie, multimorbidity) [1,2]. The coordination and integration of services for the management of patients living with multimorbidity is important for care to be efficient, safe, and less of a burden [2,3]; however, these services are often found to be suboptimal in clinical practice [4,5], which may lead to polypharmacy, increased treatment costs, side-effects, and unintended drug interactions [5]. In the end, this may overwhelm patients (eg, in what they have to do to control their disease) and in turn result in poor adherence, wasted resources, and poor outcomes [5-9].

Minimally disruptive medicine may optimize the coordination and integration of services [4]. It is a patient-centered approach to care focusing on achieving patient goals for life and health [5] by seeking care strategies that fit patient context [4]. The cumulative complexity model practically orients minimally disruptive medicine–based care [1]. In this model, the patient workload-capacity imbalance is the central mechanism driving patient complexity. Workload (“What patients have to do”) encompasses the demands on patients’ time and energy, including not only the demands of treatment and self-care, but also the demands of life in general. Capacity (“What patients can do”) concerns patients’ abilities and resources to handle health care and life work (eg, functional morbidity, financial/social resources, literacy). Workload-capacity imbalance can lead to problems accessing and using care, as well as enacting self-care [1]. Ultimately, this can result in poor adherence and poor patient outcomes [8-10].

All care strategies influence workload and capacity by affecting (positively or negatively) treatment and illness burden, respectively. For example, intensifying a patient’s treatment may reduce his symptoms and illness burden at the cost of an increased treatment burden [1]. Awareness of a patient’s capacity and workload is therefore critical in deciding on a patient’s care strategy. Indeed, inattention to contextual information may lead to errors in this choice [11].

## Shared Decision Making

When aiming for treatment decisions that result in desirable outcomes for the patient, active participation and engagement of both the clinician and patient is needed [12]. Shared decision making [13,14] is a patient-centered approach in which clinicians and patients work together to choose the best course of action for each patient’s particular situation [15]. Although shared decision making often does not impact clinical outcomes [16-18], it tends to result in improved affective and cognitive outcomes [16] and can also help facilitate a stronger clinician-patient relationship and a shared understanding of treatment for patients’ health and life goals [19,20]. Some ethical and clinical arguments also advocate for shared decision making [16,21-29], but despite this, it is not yet routine in clinical practice [30,31]. Multiple reasons are present for the suboptimal implementation of shared decision making, such as a perception-reality gap in which clinicians feel they are practicing shared decision making [31].

Shared decision making was first proposed in 1982 [32]. One of its first models was proposed by Charles et al [33,34], which later models built upon [33,34]. Since then, multiple definitions and models of shared decision making have been developed [35-39]. In this commentary, six key elements of quality shared decision making are defined [40,41].

- Situation diagnosis (understanding the patient’s situation and establishing what aspects require action)
- Choice awareness (indicating that more than one option is available and that the patient’s preferences are important in deciding on the course of action)
- Option clarification (describing the options available)
- Discussion of pros and cons (explaining the pros and cons of the available options)
- Deliberation of patient preferences (discussing the patient’s preferences)
- Making the decision [40,41]

Some shared decision making models also include patient value elicitation as a key element [42,43]. We assume that value elicitation is implicitly handled in deliberation of patient preferences, as we expect that a patient’s preferences also reflect the patient’s important values. Furthermore, the order that elements are handled within the encounter is not fixed or important as long as all elements are included. In our opinion, the natural flow of the conversation is superior to the order of the key elements or the handling of value elicitation implicitly or explicitly. Namely, the clinician and patient should work together in partnership [4,15], instead of checking the elements’ boxes in a mechanical way.

## Shared Decision Making Decision Aids

Decision aids are designed to help patients participate in decisions that involve weighing the pros and cons of different treatment options and can help patients choose an option that is congruent with their values [44]. Decision aids are designed to supplement rather than replace clinician-patient interactions [42].

The International Patient Decision Aid Standards Collaboration developed a minimum set of standards for qualifying a tool as a decision aid, which states that all shared decision making key elements, except making the decision, should be incorporated in the tool to regard it as a decision aid [45]. Despite this, most decision aids developed for chronic illnesses are focused on communicating options and their pros and cons [41]. The situation diagnosis is less often included in decision aids for persons with chronic conditions [41], while understanding the patient’s context is a prerequisite for care to fit the patient's context. Moreover, clinicians probe for contextual information less often than for biomedical information [11] and thus may neglect this information when making decisions with patients.

## Workload and Capacity Assessments

To address the problem of neglecting contextual information, we propose to systematically supplement the use of decision aids for shared decision making with workload and capacity assessments [4]. Workload assessments aim at identifying the intrusiveness of health on life and to find opportunities for treatment plan augmentation. Capacity assessments aim at identifying contextual limitations in patients’ capacity that may impact care effectiveness and that may be amenable to support or intervention [4].

The Instrument for Patient Capacity Assessment (ICAN) discussion aid is a tool that can be helpful in carrying out capacity and workload assessments [46,49]. ICAN is a paper-based tool that the patient can fill out while waiting for the clinician. It asks which areas of the patient’s life (eg, family and friends, work, living situation) are sources of satisfaction, burden, or both. The patient is also asked whether things they have been asked to do to care for their health are a help, a burden, or both. Common self-management activities are listed (eg, taking medications and self-monitoring), and blank spaces are provided for any self-management tasks not listed. During the conversation the clinician is asked to review the three questions on the back of the aid: 1) “What are you doing to manage your stress?” (brings forth typical day-to-day and competing priorities), 2) “Where do you find the most joy in your life?” (to assess if the patient is struggling with biographical disruption from their treatment and illness), and 3) “What else is on your mind today?” (focuses on the visit today) [46,49]. Biographical disruption can be described as “an assault of chronic illness on often cherished conceptions of self, identity and life course, resulting in a fundamental rethinking of one’s biography and self-concept in the light of the illness” [47,48]. The clinician then explores what the patient reported on the form by asking “What stands out to you on this sheet you filled?” [46,49]. In this way, ICAN facilitates the situation diagnosis by elucidating and sharing insights about the patient’s current workload, capacity, and treatment burden to enact treatment plans [46,49]. A Web-based implementation tool kit covering workflow integration and conversation training is now freely available [49].

## Discussion

Patients’ workloads and capacity need consideration when choosing care strategies. However, this is rarely done when performing shared decision making with patients. We thus propose to use workload and capacity assessments to add insight into the patient context more broadly than disease or decision specific decision aids.

## Abbreviations

ICAN: Instrument for Patient Capacity Assessment
